# Antihypertensive Effects of Corn Silk Extract and Its Novel Bioactive Constituent in Spontaneously Hypertensive Rats: The Involvement of Angiotensin-Converting Enzyme Inhibition

**DOI:** 10.3390/molecules24101886

**Published:** 2019-05-16

**Authors:** Chia-Cheng Li, Yu-Chen Lee, Hsin-Yi Lo, Yu-Wen Huang, Chien-Yun Hsiang, Tin-Yun Ho

**Affiliations:** 1 Graduate Institute of Chinese Medicine, China Medical University, Taichung 40402, Taiwan; u9551002@gmail.com (C.-C.L.); hsinyilo0123@gmail.com (H.-Y.L.); 2 Graduate Institute of Acupuncture Science, China Medical University, Taichung 40402, Taiwan; d5167@mail.cmuh.org.tw; 3 Department of Chinese Medicine, China Medical University Hospital, Taichung 40447, Taiwan; 4 Graduate Institute of Biomedical Sciences, China Medical University, Taichung 40402, Taiwan; cute830811@yahoo.com.tw; 5 Department of Microbiology and Immunology, China Medical University, Taichung 40402, Taiwan; 6 Department of Health and Nutrition Biotechnology, Asia University, Taichung 41354, Taiwan

**Keywords:** corn silk, hypertension, angiotensin-converting enzyme, bioactive phytopeptide

## Abstract

Corn silk tea has been used in folk medicine for anti-hypertensive healthcare. Angiotensin-converting enzyme (ACE) plays a crucial role on the homeostasis of blood pressure. However, effects of corn silk tea on ACE activity and the presence of ACE inhibitory constituents in corn silk are still unknown. Here we applied proteomics and bioinformatics approaches to identify corn silk bioactive peptides (CSBps) that target ACE from the boiling water extract of corn silk (CSE). CSE significantly reduced systolic blood pressure (SBP) levels in spontaneously hypertensive rats and inhibited the ACE activity. By proteomics coupled with bioinformatics analyses, we identified a novel ACE inhibitory peptide CSBp5 in CSE. CSBp5 significantly inhibited the ACE activity and decreased SBP levels in a dose-dependent manner. Docking analysis showed that CSBp5 occupied the substrate-binding channel of ACE and interacted with ACE via hydrogen bonds. In conclusion, we identified that CSE exhibited anti-hypertensive effects in SHRs via the inhibition of ACE, the target of most anti-hypertensive drugs. In addition, an ACE inhibitory phytopeptide CSBp5 that decreased SBP levels in rats was newly identified. Our findings supported the ethnomedical use of corn silk tea on hypertension. Moreover, the identification of ACE inhibitory phytopeptide in corn silk further strengthened our findings.

## 1. Introduction

Hypertension is the most common serious chronic health problem in the world. The World Health Organization reports that increased blood pressure affects 1.13 billion people worldwide and the prevalence in females aged ≥18 years was around 20% and in males around 24% in 2015. Moreover, hypertension is a major risk factor for the development of stroke, heart failure, coronary artery disease, and renal failure [1]. Renin-angiotensin-aldosterone system (RAAS) is a well known mechanism that controls the blood pressure by regulating the volume of fluid in the body. Angiotensin-converting enzyme (ACE) is a crucial dipeptidyl carboxypeptidase in RAAS, that converts angiotensin I to the active vasoconstrictor angiotensin II [2]. ACE consists of two catalytically active domains (N- and C-domains), which display differences in substrate processing abilities. The C-domain of ACE hydrolyzes hippuryl-l-histidyl-l-leucine (H-HL) at a faster rate (9-fold) compared to the N-domain, while both domains hydrolyze *N*-benzyloxycarbonyl-l-phenyl- alanyl-l-histidyl-l-leucine (Z-FHL) at an equal rate [3]. Inhibition of ACE activity decreases the production of angiotensin II, leading to vasodilation and reduced blood pressure. Therefore, ACE inhibitors, such as captopril, lisinopril and enalapril, are widely used as pharmaceutical drugs for the treatment of hypertension [4].

Food peptides with anti-ACE activities have been identified from plants and animals [5,6]. For example, oligopeptides FDGIP and AIDPVRA are ACE inhibitor derived from *Caulerpa lentillifera* [7]. ACE inhibitory peptides released from alcalase-hydrolyzed amaranth protein exhibit hypotensive effects in spontaneously hypertensive rats (SHRs) [8]. ACE inhibitory peptide GAAGGAF from *Coix* glutelin has a significant antihypertensive effect [9]. Peptides from auto-digested reishi (*Ganoderma lingzhi*) extract show potent inhibition against ACE and hypotensive activities in SHRs [10]. Lacto-tripeptides Val-Pro-Pro (VPP) and Ile-Pro-Pro (IPP), identified from sour milk, inhibit the ACE activity and protect the endothelial function in vitro, and exhibit anti-hypertensive effects in SHRs [11]. Clinical trials on subjects with blood pressure ranging from normal to mild hypertension show that intake of tablets containing VPP and IPP leads to a mild improvement in hypertension without side effects [12]. These results suggest that the presence of food-derived ACE inhibitory peptides makes foods show anti-hypertensive potentials.

Corn or maize (*Zea mays*), one of the most important crops in the world, has become a staple food in many parts of the world. Corn silk is the shiny thread-like fibers that grow as part of ears of corn. Corn silk tea is an herbal remedy that has been used in folk medicine for healthcare applications, such as relieving inflammation, edema, hyperlipidemia, hyperglycemia, hypertension, and obesity in China, Korea, Vietnam, America, and some other countries [13,14]. Corn silk contains a variety of pharmacologically-active compounds. For example, flavonoids of corn silk exhibit anti-diabetic, anti-oxidant, and anti-hyperlipidemic activities on diabetic mice [15,16]. Corn silk aqueous extract, containing alkaloids, flavonoids, phenols, saponins, tannins and phytosterols, inhibits α-amylase and α-glucosidase activities, and provides an effective strategy to modulate levels of postprandial hyperglycemia via control of starch metabolism [17]. PSC2, a hetero- polysaccharide from corn silk, decreases blood glucose and serum insulin levels, and improves glucose intolerance in type 2 diabetic mice [18]. FK2, the peptide from trypsin hydrolysis of corn silk, inhibits nuclear factor-κB activation and ameliorates bacterial endotoxin-induced acute systemic inflammation in mice [19]. Anti-hypertensive effect of corn silk has been reported previously [20]. Recent study conducting systematic review and meta-analysis of randomized controlled trials also showed that corn silk tea plus antihypertensive drugs could be more effective on lowering blood pressure than conventional antihypertensive drugs alone [21]. Corn silk extract reduces intraocular pressure in systemic and non-systemic hypertensive subjects. Moreover, the reduction of blood pressure by corn silk is due to potassium-induced natriuresis and diuresis [20]. However, effects of corn silk on the ACE activity and the presence of ACE inhibitory constituents in corn silk have not been studied so far.

Here we evaluated the effects of corn silk tea on ACE activity in vitro and hypertension in SHRs. Captopril, lisinopril, and enalapril, the well-known ACE inhibitors, are amino acid or dipeptide drugs. We hypothesized whether there were ACE inhibitory peptides present in corn silk extract (CSE). Proteomics and bioinformatics approaches were therefore applied to identify anti-hypertensive peptides in corn silk tea. Peptide sequences were identified using liquid chromatography coupled with tandem mass spectrometry (LC-MS/MS). A combination of bioinformatics tools, such as PeptideRanker and BIOPEP, was applied to select peptides with potential bioactivities. ACE activity assay and spontaneously hypertensive animal model were applied to evaluate the anti-hypertensive effects of peptides. Furthermore, molecular docking was carried out to clarify the interaction between ACE and anti-hypertensive peptides in corn silk. Our data showed that the boiling water extract of corn silk inhibited the ACE activity and exhibited anti-hypertension effects in SHRs. In addition, we newly identify an ACE inhibitory phytopeptide in corn silk that interacted with the substrate-binding channel of ACE and consequently decreased systolic blood pressure (SBP) levels in rats.

## 2. Results

### 2.1. CSE Reduced Blood Pressure In Vivo and Inhibited ACE Activity In Vitro

**Figure 1:**
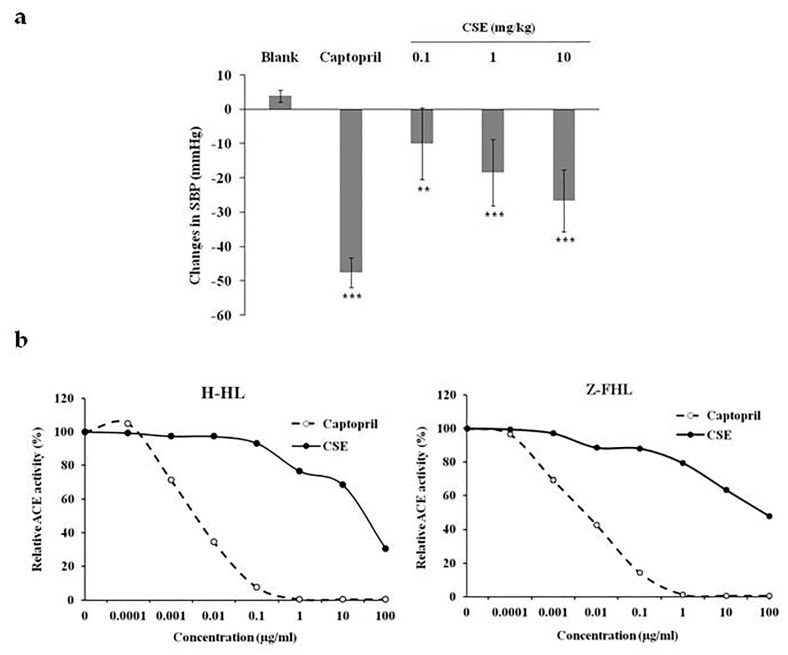
Effects of CSE on blood pressure and ACE activity. (**a**) Anti-hypertensive effects of CSE in rats. SHRs were orally given with 10 mg/mL captopril or various dosages of CSE. Tail SBP was measured at 0 and 1 h. Data are expressed as changes in SBP (mmHg). Values are mean ± standard error (*n* = 6). ** *p* < 0.01, *** *p* < 0.001, compared with blank. (**b**) ACE activity assay. Various amounts of captopril or CSE were mixed with serum ACE and substrates (H-HL or Z-FHL). The hydrolyzed substrates were then labeled with fluorescence and measured using a fluorometer. Data are expressed as relative ACE activity (%), which is presented as the comparison with the fluorescence relative to blank. Values are mean ± standard error (*n* = 6).

To analyze whether CSE exhibited anti-hypertensive effects, we treated SHRs with 10 mg/kg captopril or various amounts of CSE orally. Tail SBP was measured at 0 and 1 h. As shown in Figure 1a, the SBP level of captopril group was 47.6 ± 4.34 mmHg lower than that of the blank group. Oral administration of CSE significantly reduced the SBP levels in a dose-dependent manner. The SBP level of 10 mg/kg CSE was 26.57 ± 9.03 mmHg lower than that of the blank group.

We further analyzed the effects of CSE on the ACE activity using H-HL and Z-FHL substrates. As shown in Figure 1b, captopril inhibited the hydrolysis of H-HL and Z-FHL substrates by ACE in a dose-dependent manner. CSE also suppressed the ACE activity in a dose-dependent manner. The hydrolysis of H-HL and Z-FHL substrates by ACE was inhibited by CSE, with IC_50_ values of 53.95±0.58 μg/mL and 87.26±2.68 μg/mL, respectively. These findings suggested that CSE inhibited the ACE activity and sequentially reduced SBP levels in rats.

### 2.2. Identification of Bioactive Peptides with Blood Pressure Lowering-Potentials in CSE

**Figure 2:**
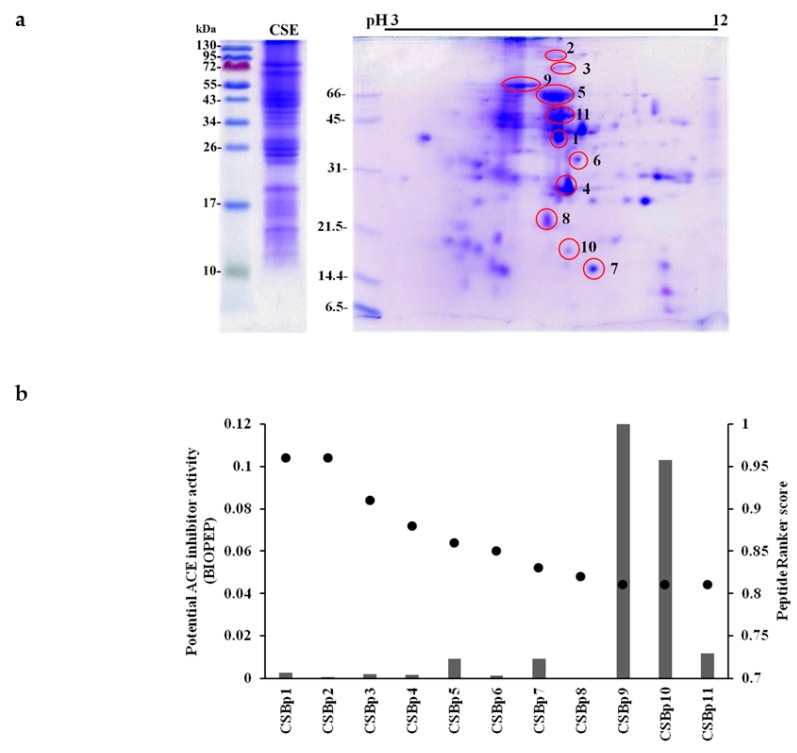
Analysis of potential bioactive peptides in CSE. (**a**) Protein profiles of CSE. Proteins in CSE were separated by SDS-PAGE (left) and 2-DE (right) on 15% polyacrylamide gels. Proteins were visualized by Coomassie Brilliant Blue R-250. Protein spots in red circles were further analyzed by LC-MS/MS. Protein size markers (in kDa) are shown at the left. Photographs are representative images of three independent experiments. (**b**) Prediction of potential bioactive peptides using PeptideRanker and BIOPEP. Dots represent PeptideRanker scores. Bars represent potential ACE inhibitor activity analyzed by BIOPEP.

The well-known ACE inhibitors, such as captopril, lisinopril and enalapril, are amino-acid or dipeptide drugs. Moreover, CSE was a protein-rich extract and the protein content of CSE was 10.86%. Therefore, we analyzed protein profiles of CSE by sodium dodecyl sulfate-polyacrylamide gel electrophoresis (SDS-PAGE) and two-dimensional electrophoresis (2-DE). As shown in Figure 2a, CSE was composed of various proteins, with the molecular weight ranging from 10 to 100 kDa. 2-DE analysis showed that there were several visible protein spots and some smears in the gels. Eleven protein spots with neutral pH were further excised from stained gels, digested by gastrointestinal protease (trypsin), and identified using LC-MS/MS analysis. The identified protein spots of CSE are summarized in Table 1.

A total of 133 peptide spectra matched to aforementioned proteins was screened using PeptideRanker. Top 10% of peptides, which had cut-off scores of approximately 0.8, were considered as potential corn silk bioactive peptides (CSBps) (Figure 2b). The MS data of these peptides were shown in Table 2. These peptides were further subjected to BIOPEP database to calculate the potential ACE inhibitor activity. Top 5 peptides with potential ACE inhibitor activity were CSBp9 (GLIYPPFSNIR), CSBp10 (EPFIRPPR), CSBp11 (MNVPPGPFMAR), CSBp5 (SKFDNLYGCR), and CSBp7 (AMPTFFLIK).

Docking analysis was further performed to evaluate the interaction between ACE and CSBps. As shown in Table 2, CSBp5 had a highest docking score, followed by CSBp1, CSBp7, and CSBp11. CSBp9 displayed a largest interface area of peptide-ACE complex, followed by CSBp5, CSBp1, and CSBp10. Because CSBp5 displayed a potential ACE inhibitor activity and has a highest docking score with ACE interaction, CSBp5 was synthesized for in vitro ACE activity assay and in vivo hypertensive assay.

### 2.3. CSBp5 Inhibited ACE Activity In Vitro and Reduced Blood Pressure In Vivo

Various amounts of CSBp5 were mixed with mouse sera in the presence of H-HL or Z-FHL substrate, and the ACE activity was determined by measuring the fluorescence-labeled hydrolyzed substrates.

**Figure 3:**
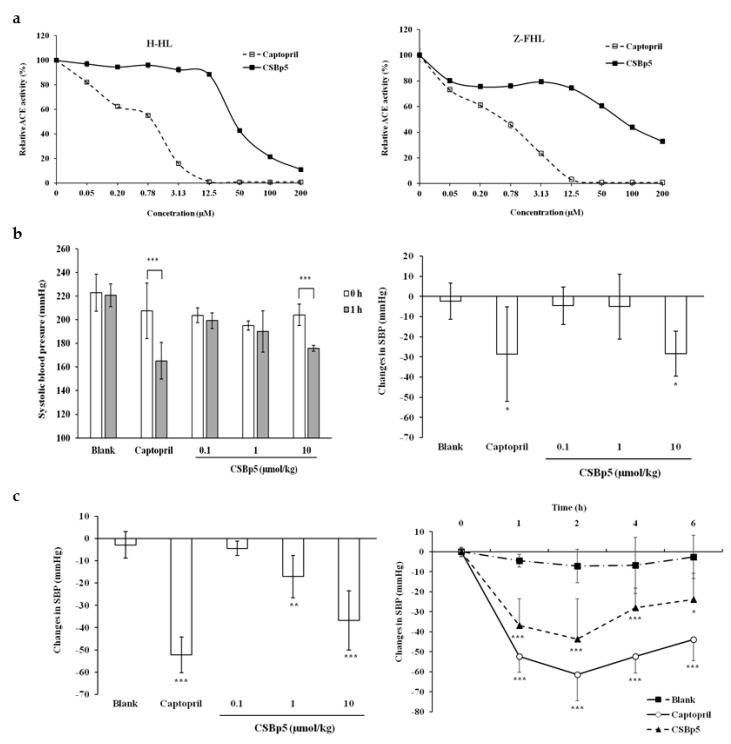
Effects of CSBp5 on ACE activity and blood pressure. (**a**) ACE activity assay. Various amounts of captopril or CSBp5 were mixed with serum ACE and substrates (H-HL (top panel) or Z-FHL (bottom panel)). The mixtures were incubated at 37 °C for 20 min, and the resulting products were labeled with fluorescence and measured using a fluorometer. Data are expressed as relative ACE activity (%), which is presented as the comparison with the fluorescence relative to blank. Values are mean ± standard error (*n* = 6). (**b**) Anti-hypertensive effect of CSBp5 by intraperitoneally injection. SHRs were intraperitoneally given with 10 μmol/kg captopril or various dosages of CSBp5. Tail SBP was measured at 0 and 1 h. Data are expressed as SBP (mmHg) (top panel) or changes in SBP (mmHg) (bottom panel). Values are mean ± standard error (*n* = 6). * *p* < 0.05, ** *p* < 0.01, *** *p* < 0.001, compared with SBP at 0 h (top panel) or with blank (bottom panel). (**c**) Anti-hypertensive effect of CSBp5 by oral administration. SHRs were orally given with 10 μmol/kg captopril, various dosages of CSBp5 (top panel), or 10 μmol/kg CSBp5 (bottom panel). Tail SBP was measured at 0 and 1 h (top panel), or at indicated time point (bottom panel). Data are expressed as changes in SBP (mmHg). Values are mean ± standard error (*n* = 6). * *p* < 0.05, ** *p* < 0.01, *** *p* < 0.001, compared with blank.

As shown in Figure 3a, captopril inhibited the hydrolysis of both H-HL and Z-FHL substrates by ACE in a dose-dependent manner. The IC_50_ values of captopril were 0.68 ± 0.09 µM (H-HL substrate) and 1.08 ± 0.09 µM (Z-FHL substrate). CSBp5 also inhibited the ACE activity and the inhibition displayed a dose-dependent manner. The IC_50_ value of CSBp5 for the hydrolysis of H-HL by ACE was 44.11 ± 1.04 μM, while IC_50_ value for the hydrolysis of Z-FHL was 81.71 ± 1.06 μM. Moreover, our findings suggested that CSBp5 exhibited a more effective inhibition on the C-domain of ACE. Anti-hypertensive effects of CSBp5 were further evaluated by administering (intraperitoneally) various amounts of CSBp5 in SHR. Tail SBP was measured 1 h after injection. As shown in Figure 3b, a slight decrease of SBP was observed 1 h later in blank group, while a significant decrease (52.83±19.06 mmHg) was observed in captopril group. Intraperitoneal injection of CSBp5 decreased SBP levels in a dose-dependent manner. A significant decrease (28.33 ± 12.5 mmHg injection) was observed in 10 μmol/kg CSBp5 group. Oral administration of CSBp5 also decreased the SBP levels in a dose-dependent manner and a significant decrease (36.78 ± 13.25 mmHg) was observed in 10 μmol/kg CSBp5 group (Figure 3c). Time-course effect of anti-hypertensive ability of CSBp5 was further evaluated in SHR. SHRs were orally given with 10 μmol/kg captopril or CSBp5, and the tail SBP was measured at 0, 1, 2, 4, and 6 h. As shown in Figure 3c, oral administration of water (blank) did not affect SBP levels. However, administration of captopril and CSBp5 caused a significant decrease in SBP, with SBP falling by approximately 60 mmHg and 30 mmHg, respectively, at 2 h. Moreover, the decrease SBP level by CSBp5 was persistent during a 6-h measurement. The changes in diastolic blood pressures were also consistent with the changes in SBP (Supplementary Materials Figure S1). Therefore, these data suggested that CSBp5 inhibited ACE activity and decreased SBP levels in rats.

### 2.4. Interaction between CSBp5 and ACE by Docking Analysis

The possible ACE inhibitory mechanism of CSBp5 was investigated by molecular docking in silico. ACE comprises two similar protein domains (N- and C-domains) and C-domain of ACE is mainly responsible for angiotensin II formation [22]. In addition, our data showed that the selectivity of CSBp5 for C-domain versus N-domain was approximately 2-fold. The crystal structure of C-domain of human ACE (PDB ID: 4APH) was therefore applied as a target for docking analysis.

**Figure 4:**
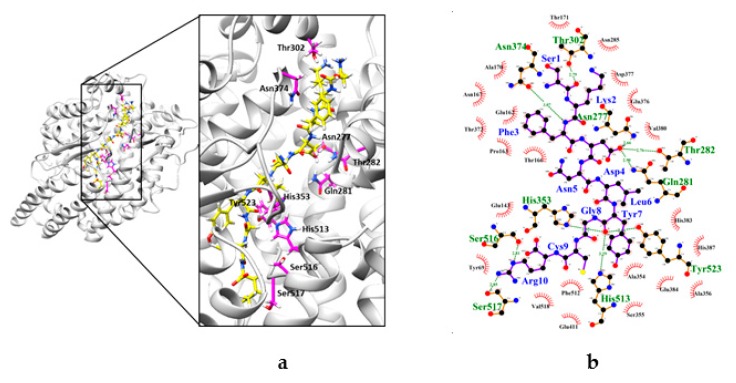
Interaction between CSBp5 and ACE by docking analysis. (**a**) Docking structure of ACE complexed with CSBp5. Close-up view of ACE complexed with CSBp5 is shown on the right panel. The structure of CSBp5 and ACE are represented by yellow stick and white ribbon, respectively. The side chains of residues forming hydrogen bonds with CSBp5 are shown as pink sticks. (**b**) The LigPlus schematic 2D representation of CSBp5-ACE interaction. The amino acid residues of CSBp5 are shown as purple sticks and labeled in blue. Hydrogen bonds between CSBp5 and ACE are represented by green dashed lines. The amino acid residues of ACE interacted with CSBp5 are shown as brown sticks and labeled in green.

As shown in Figure 4, CSBp5 occupied the substrate-binding channel of ACE. Moreover, CSBp5 formed potential interactions with residues Asn277, Gln281, Thr282, Thr302, His353, Asn374, His513, Ser516, Ser517, and Tyr523 of ACE through hydrogen bonds. Four residues, including Gln281, His353, His513 and Tyr523, were commonly observed among CSBp5-ACE and angiotensin II-ACE interaction [23]. The distance between Gln281 of ACE and Asp4 of CSBp5 was 2.99 Å, while the distances between His353, His513, and Tyr523 of ACE and Gly8 CSBp5 were 2.85, 3.29, and 3.06 Å, respectively. These findings suggested that CSBp5 might interact with the substrate-binding channel of ACE and prevent angiotensin I from entering the catalytic pocket of ACE.

## 3. Discussion

Although corn silk has been used as an aqueous decoction for anti-hypertensive healthcare in folk medicine, there are few reported studies on the certification of anti-hypertensive effects and mechanisms of corn silk in human subjects or animal models. Martín et al. [24] reported that intravenous injection of 1.342 mg/kg boiling dialysate of corn silk decreases diastolic blood pressure by 63.8% ± 33.6% in normotensive anaesthetized dogs. George and Idu [20] reported that oral administration of 260 mg/kg corn silk aqueous extract reduces the intraocular pressure in eyes with ocular hypertension and lowers the blood pressure in systemic and non-systemic hypertensive subjects. Because the high potassium content is observed in the corn silk extract, and diuretic and uricosuric properties have been discovered in corn silk, they proposed that the fall in blood pressure by corn silk may result from the diuretic activity or direct vasodilatation [25]. Anti-hypertensive activities of CSE were analyzed in SHRs by tail-cuff method in this study. Telemetric blood pressure monitoring system is a direct and effective method for measuring blood pressure of laboratory animals. However, implantation of a radio transmitter is required for this method. Moreover, the cost of a telemetry apparatus is quite high. Tail-cuff sphygmomanometer has been used as an indirect method to monitor blood pressure in rats for many years. Although multiple factors, such as stress and temperature, may affect the measurement, previous report indicated that tail-cuff method is as good as telemetry for measuring blood pressure in the conscious rats [26]. We found that corn silk extract displayed anti-hypertensive effects in SHRs. Moreover, in vitro serum ACE activity was inhibited by corn silk in a dose-dependent manner. These findings suggested that corn silk exhibited anti-hypertensive abilities via the inhibition of ACE, the target of anti-hypertensive pharmaceutical drugs.

CSE was a protein-rich extract and the protein content of CSE was 10.86%. Moreover, the well-known ACE inhibitors are amino acid or dipeptide drugs. Therefore, we proposed that there were bioactive peptides responsible for the anti-hypertensive activities of CSE. Proteomics and bioinformatics analyses were further applied to identify the anti-hypertensive bioactive peptides in CSE. Peptides with anti-hypertensive potentials have been determined from corn. For example, dipeptide Ala-Tyr (AY) has been identified from corn gluten meal, a major byproduct of corn wet milling. AY affects the ACE activity with an IC_50_ of 14.2 μM and a maximal reduction (9.5 mmHg) of SBP is observed 2 h after oral administration of 50 mg/kg AY in SHRs [27]. Several corn peptides have been identified from zein, an alcohol soluble protein present in corn gluten meal. Tripeptide (Leu-Arg-Pro, Leu-Ser-Pro, and Leu-Gln-Pro), identified from thermolysin-hydrolyzed zein, inhibited the ACE activity in vitro with IC_50_ values of 0.25–1.9 μM. Moreover, thermolysin hydrolysate (5 g/kg) of zein reduces SBP (14 mmHg) at 6 h after oral administration in SHRs [28]. Oral administration of corn peptides (10 mg/kg) from hydrolyzed zein decreased SBP (42.5 mmHg) in SHRs at 8 h. Further HPLC-MS/MS analysis identified that a tetrapeptide Met-Ile/Leu-Pro-Pro exhibited effective ACE inhibitory activities with an IC_50_ of 70.32 μg/mL (155 μM) in vitro [29]. Interestingly, we did not find the presence of these corn peptides in the aqueous extract of corn silk by LC-MS/MS analysis. This might be because the previously reported peptides were from protein digests or the previously reported extract was not like the sample used in this work. Moreover, our data showed that CSBp5 inhibited the ACE activity with an IC_50_ value of 44.11 ± 1.04 μM and decreased SBP levels (28.33 ± 12.5 mmHg injection; 36.78 ± 13.25 mmHg oral) at 1 h after administration of 10 μmol/kg (12 mg/kg) CSBp5. Therefore, our study first identified the presence of anti-hypertensive peptide (CSBp5) in corn silk. Furthermore, in comparison of ACE inhibitory peptides in corn, our findings suggested that CSBp5 showed more effective ACE inhibitory activity and anti-hypertensive abilities in SHRs. Moreover, our study showed that CSBp5, like captopril, was an ACE inhibitory peptide. ACE inhibition has been demonstrated in both healthy human subjects and animals by showing that the elevation of blood pressure caused by exogenously administered angiotensin I was attenuated or abolished by captopril. Therefore, we expect that CSBp5 may reduce angiotensin I-induced SBP in normal animal.

CSBp5 was an ACE inhibitory peptide that exhibited a more effective inhibition on the C-domain of ACE. Somatic ACE is composed of two similar catalytic domains (N- and C-domains). Both domains are efficient for the cleavage of angiotensin I. However, RXP407, an ACE N-domain selective inhibitor, displays no effect on blood pressure, suggesting that C-domain is the dominant site for angiotensin II formation [22,23]. Moreover, previous study indicated that C-domain selective ACE inhibitors are likely to reduce blood pressure by lowering angiotensin II and have improved side effect profiles [30]. Docking analysis was therefore performed to elucidate the interaction between CSBp5 and C-domain of ACE. The crystal structure of human C-domain somatic ACE in complex with angiotensin II has been elucidated [23]. The co-crystal structure showed that angiotensin II (Asp-Arg-Val-Tyr-Ile-His-Pro-Phe) can be observed in the substrate-binding channel of ACE. The main interacting residues at the ACE active site are divided into three substrate binding pockets S1 (Ala354, Glu384 and Tyr523), S2’ (Gln281, Tyr520, Lys511, His513 and His353), and S1’ (Glu162) [31]. Ile5 and His6 residues of angiotensin II interact with Ala354 residue of S1 pocket and all the active site residues of S2’ pocket [23]. In this study, we found that CSBp5 (Ser-Lys-Phe-Asp- Asn-Leu-Tyr-Gly-Cys-Arg), like angiotensin II, was located in the substrate-binding channel of ACE. CSBp5 exhibited hydrogen bonds with 10 amino acid residues of ACE. Asp4 and Tyr7 residues of CSBp5 interacted with Tyr523 residue of S1 pocket, and Gln281, His513, and His353 active site residues of S2’ pocket. Therefore, we propose that CSBp5 might be a competitive inhibitor by preventing substrates from binding to the active sites of ACE. Nevertheless, proteins are usually digested to amino acid residues or small peptides by pepsin in the stomach and pancreatin in the small intestine after ingestion. Endocytosis, phagocytosis, transcytosis, direct penetration, and paracellular transport are usually responsible for the transport of peptides in the intestinal epithelial [32]. The detailed gastrointestinal transport mechanism of CSBp5 after ingestion remained to be further elucidated. In addition, short half-life is one of the key challenges of therapeutic peptides. Peptide database covering 1,193 unique peptides shows that the half-life of these peptides ranges from 60 sec to 86,400 sec [33]. The exact half-life of CSBp5 needs to be verified by further experiments.

## 4. Materials and Methods

### 4.1. Chemicals

All chemicals, except indicated, were purchased from Sigma-Aldrich (St. Louis, MO, USA). ACE substrates, including H-HL (Catalog No. M-1485) and Z-FHL (Catalog No. M-1305), were purchased from Bachem (Bubendorf, Switzerland). H-HL and Z-FHL were dissolved at 100 mM in 80% acetic acid and in 100% methanol, respectively.

### 4.2. Plant Materials and Extraction Procedure

Maize plants (*Zea mays* L. var. *rugosa* Bonaf.) were harvested from corn fields in Taichung city, Taiwan. Corn silk (*Stigma maydis*) at milky stage of corns was collected in April 2017 and authenticated at Department of Chinese Pharmaceutical Sciences and Chinese Medicine Resources, China Medical University. Voucher specimen has been deposited at LiFu Museum of Chinese Medicine, China Medical University (Taichung, Taiwan). CSE was prepared by mixing 10 g of fresh corn silk with 30 mL of distilled water at 100 °C for 30 min. The supernatant was then collected by centrifugation and stored at −20 °C for further analysis. Protein concentration was quantified by a Bradford method (Bio-Rad, Hercules, CA, USA; Catalog No. 500-0006).

### 4.3. Animal Experiments

Male SHRs (200–250 g, 10 week-old) were purchased from National Laboratory Animal Center (Taipei, Taiwan). Rats were maintained under a 12 h day/12 h night cycle and had free access to water and food. Rat experiments were conducted under ethics approval from China Medical University Animal Care and Use Committee (Permit No. 104-75-N).

SHRs with tail SBP ≥ 180 mmHg were randomly divided into five groups with six rats per group. Rats were given intraperitoneally or orally with 400 μL of distilled water (blank), captopril (Catalog No. C4042), or various dosages of CSE or peptide (dissolved in water). SBP was measured by a tail-cuff apparatus (Non-Invasive Blood Pressure System, Panlab, Barcelona, Spain). Rats were placed in a warm holder kept at 37 ± 1 °C for 10 min before the measurement. Changes in SBP were calculated by subtracting SBP at indicated time point from SBP at 0 h.

### 4.4. ACE Activity Assay

ACE activity assay was performed as described previously with a slight modification [34]. Mouse sera collected from normal BALB/c mice were used as the source of soluble somatic ACE. ACE activity in mouse serum was determined as described previously [35]. One unit of ACE converts 1 μmol HL/min at 37 °C and the ACE unit in mouse serum was 2 U/μL. A 25-μL reaction mixture, containing 2 µL mouse serum, indicated concentrations of inhibitors (captopril, CSE, or peptide), 400 mM boric acid (pH 8.3) and 300 mM sodium chloride, were incubated at 37 °C for 20 min. Ten microliters of 7 mM H-HL or 3.5 mM Z-FHL were added and the mixture was incubated at 37 °C for 2 h. The ACE activity was then stopped by the addition of 150 μl of 0.34 M NaOH. The free N-terminus of product HL was fluorogenically labeled with *o*-phthaldialdehyde (OPA, Catalog No. P1378) by adding 20 µl of 20 mg/mL OPA and incubating at room temperature for 10 min. The reaction was then stopped by the addition of 50 μL of 3 M HCl and the fluorescent product was measured using a fluorometer (Fluoroskan Ascent FL, Thermo Fisher, Waltham, MA, USA) with an excitation wavelength at 355 nm and an emission wavelength at 535 nm. To correct the intrinsic fluorescence of mouse serum, blank was performed according to the aforementioned procedures without inhibitors. Relative ACE activity (%) was calculated as:

(fluorescence intensity of inhibitor/fluorescence intensity of blank) × 100.(1)

IC_50_ represents the concentration of the drug that is required for 50% inhibition in vitro.

### 4.5. 2-DE and LC-MS/MS Analysis

Protein components of CSE were identified by 2-DE and LC-MS/MS as described previously [36]. Briefly, CSE (200 μg protein) was applied to IPG strips (7 cm, pH 3–10) and isoelectric focusing was performed using a Protean IEF Cell (Bio-Rad, Hercules, CA, USA). Focused IPG strips were separated by SDS-PAGE and protein spots on the gels were visualized by Coomassie Brilliant Blue R-250. Protein spots were then excised from stained gels, digested by trypsin, and identified using an Ultimate capillary LC system (LC Packings, Amsterdam, The Netherlands) coupled to a QSTAR^®^ XL quadrupole-time-of-flight mass spectrometer (Applied Biosystem/MDS Sciex, Foster City, CA, USA), which was served by Biotechnology Center, National Chung Hsing University (Taichung, Taiwan) [37]. MS/MS data were matched against green plant (Viridiplantae) taxonomy using the MASCOT search program [38]. Protein score (Table 1) reflects the combined scores of all observed mass spectra that can be matched to amino acid sequences within that protein. In general, a higher score indicates a more confident match. Peptide score (Table 2) is a measure of how well the observed MS/MS spectrum matches to the stated peptide. In this study, ion scores >38 indicate homology and ion scores >50 indicate identity (*p* < 0.05) [38].

### 4.6. Bioinformatics Analysis

Peptides identified by LC-MS/MS were screened by PeptideRanker, a bioinformatics tool for the prediction of bioactive peptides based on a novel N-to-1 neural network [39]. Peptides with the score ≥ 0.8 were selected as potential bioactive peptides and further subjected to BIOPEP database to calculate the potential ACE inhibitor activity of peptide [40].

### 4.7. Preparation of Synthetic Peptides

Peptides were synthesized in solid phase using Fmoc and Noc chemistry (LifeTein, Somerset, NJ, USA). The purities and the amino acid sequences of peptides were identified by high-performance liquid chromatography and MS, respectively. The peptides with purities ≥ 95% were used in this study.

### 4.8. Docking Analysis

Docking analysis was performed by PatchDock [41]. The crystal structure of C-domain of human ACE (PDB ID: 4APH) was obtained from protein data bank. Chain A of 4APH structure was chosen as a target using the target selection tab in PatchDock. The structures of peptides were generated using PEPstrMOD [42] and saved as a pdf format. The pdb files were uploaded using the ligand molecular selection tab in PatchDock. Enzyme-inhibitor complex type was selected to restrict the search space in the cavities of ACE structure. The binding mode and binding affinity were evaluated by the geometric shape complementarity score. All docking structures were generated by UCSF Chimera [43]. The interaction of residues between peptide and ACE was analyzed by LigPlot [44].

### 4.9. Statistical analysis

Data were presented as mean ± standard error. Data were analyzed by one-way ANOVA and post hoc Bonferroni test using SPSS Statistics v20 (IBM, Armonk, NY, USA). A *p*-value < 0.05 was considered as statistically significant

## 5. Conclusions

Corn silk has no significant adverse effects in rats orally given corn silk extract for 90 consecutive days [45]. Subchronic oral administration of corn silk extract shows the no-observed-adverse-effect level is 10 g/kg/day for both male and female mice. Moreover, micronucleus assay and sperm malformation assay showed corn silk is not a genotoxic substance [46]. These studies suggested that corn silk tea is an herbal remedy with low toxicity. In this study, we identified that the boiling water extract of corn silk exhibited anti-hypertension effects in SHRs via the inhibition of ACE, the target of anti-hypertensive drugs. In addition, an ACE inhibitory phytopeptide CSBp5 that decreased SBP levels in rats was identified in corn silk. Therefore, our findings provided a reasonable explanation on why corn silk tea displays anti-hypertensive effects in folk medicine. Moreover, the identification of ACE inhibitory phytopeptide in corn silk extract further strengthened our findings.

## Figures and Tables

**Table 1:** CSE proteins identified by LC-MS/MS.

Spot Number	Protein Name	Accession Number	Length	Molecular Weight (Da)	Score ^1^	PSM ^2^	Coverage (%) ^3^
1	Acidic endochitinase	ONM51744	308	33,748	274	17	31.82
2	Lipoxygenase	NP_001105003	873	98,164	206	32	4.81
3	Heat shock 70 kDa protein 4	ACG43420	848	93,569	166	7	5.90
4	Ascorbate peroxidase	ACO90192	250	27,597	78	8	8.00
5	Adenosylhomocysteinase	NP_001148534	485	53,248	349	22	22.06
6	APx3-Peroxisomal ascorbate peroxidase	NP_001148710	290	32,072	67	7	2.76
7	Uncharacterized LOC100216603	NP_001336786	129	14,353	45	3	15.50
8	60S Ribosomal protein L37a-2	ONM18155	194	21,777	152	5	12.37
9	NADP-dependent malic enzyme	ACX50497	608	67,164	154	15	9.87
10	Trypsin inhibitor precursor	NP_001152433	175	19,060	120	6	17.14
11	Cytochrome P450 CYP74A19	ACG28578	483	53,105	96	11	13.67

^1^ The sum of MASCOT MS/MS ion scores of all peptides that were identified. ^2^ The total number of identified peptide spectra matches for the protein. ^3^ The percentage of the protein sequence covered by identified peptides.

**Table 2:** LC-MS/MS and docking parameters of corn silk peptides.

Peptide ^1^	Amino Acid Sequence	Molecular Weight (Da)	Ion Score ^2^	Mass Error (ppm)	Docking Score ^3^	Area ^4^
CSBp1	CGFPPAGYLRR	1293	24	−1.470	10,718	1569.4
CSBp2	DAPWWPK	898	31	0.557	7890	1018.7
CSBp3	DLASFPFR	951	37	0.420	9928	1215.1
CSBp4	NCAPLMLR	989	27	0.616	9788	1429.9
CSBp5	SKFDNLYGCR	1258	65	0.715	10,872	1498.7
CSBp6	NCAPIMLR	989	27	−6.670	8938	1111.3
CSBp7	AMPTFFLIK	1066	11	0.554	10,596	1438.5
CSBp8	YFCEFCGK	1109	36	0.811	8318	1203.8
CSBp9	GLIYPPFSNIR	1275	56	0.784	9930	1630.6
CSBp10	EPFIRPPR	1010	31	1.880	9828	1479.5
CSBp11	MNVPPGPFMAR	1215	58	−3.537	10,428	1380.1

^1^ Table shows the peptides listed in Figure 2b. ^2^ Ion Score is a measure of how well the observed MS/MS spectrum matches to the stated peptide. ^3^ Score: Geometric shape complementarity score. ^4^ Area: Approximate interface area of the complex.

## Supplementary Materials

The following are available online at https://www.mdpi.com/1420-3049/24/10/1886/s1, Figure S1: Effects of CSBp5 on diastolic blood pressure.
