# Supplementary material for: Antihypertensive Effects of Corn Silk Extract and Its Novel Bioactive Constituent in Spontaneously Hypertensive Rats: The Involvement of Angiotensin-Converting Enzyme Inhibition
Source: Molecules. 2019 May 16;24(10):1886. doi: 10.3390/molecules24101886 (PMC6572293; doi:10.3390/molecules24101886)
Supplement: Supplementary file 1 [file molecules-24-01886-s001.pdf]

Supplementary Material

Antihypertensive Effects of Corn Silk Extract and Its Novel Bioactive Constituent in Spontaneously Hypertensive Rats: The Involvement of Angiotensin-Converting Enzyme Inhibition

Chia-Cheng Li [1], Yu-Chen Lee [2,3], Hsin-Yi Lo [1], Yu-Wen Huang [4], Chien-Yun Hsiang [5,*] and Tin-Yun Ho [1,6,*]

1 Graduate Institute of Chinese Medicine, China Medical University, Taichung 40402, Taiwan; u9551002@gmail.com (C.C.L.); hsinyilo0123@gmail.com (H.Y.L.)
2 Graduate Institute of Acupuncture Science, China Medical University, Taichung 40402, Taiwan; d5167@mail.cmuh.org.tw
3 Department of Chinese Medicine, China Medical University Hospital, Taichung 40447, Taiwan
4 Graduate Institute of Biomedical Sciences, China Medical University, Taichung 40402, Taiwan; cute830811@yahoo.com.tw
5 Department of Microbiology and Immunology, China Medical University, Taichung 40402, Taiwan
6 Department of Health and Nutrition Biotechnology, Asia University, Taichung 41354, Taiwan

* Correspondence: cyhsiang@mail.cmu.edu.tw (C.Y.H.); tyh@mail.cmu.edu.tw (T.Y.H.)

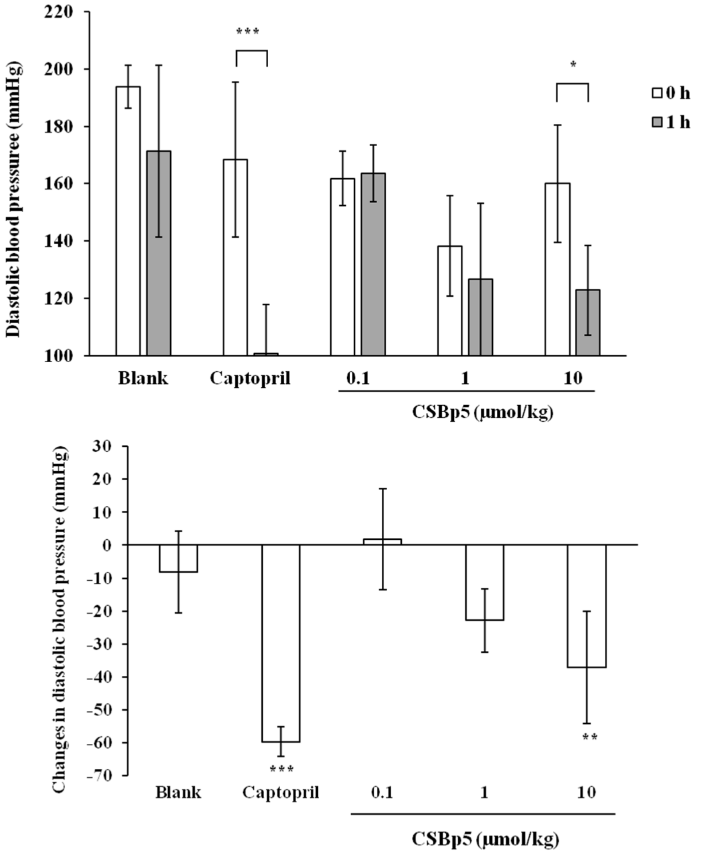

Figure S1. Effects of CSBp5 on diastolic blood pressure. SHRs were orally given with 10 μmol/kg captopril or various dosages of CSBp5. Tail diastolic blood pressure was measured at 0 and 1 h. Data are expressed as diastolic blood pressure (mmHg) (top panel) or changes in diastolic blood pressure (mmHg) (bottom panel). Values are mean ± standard error (n=6). **p*< 0.05, ***p*< 0.01, ****p* < 0.001, compared with SBP at 0 h (top panel) or with blank (bottom panel).
